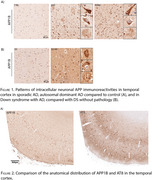# Intracellular Amyloid Precursor Protein pathology: an unrecognized neuropathological hallmark of Alzheimer's disease

**DOI:** 10.1002/alz70855_104310

**Published:** 2025-12-24

**Authors:** Sonia Sirisi Dolcet, Érika Sánchez‐Aced, Olivia Belbin, Oriol Dols‐Icardo, Maria Carmona‐Iragui, Daniel Alcolea, Sergi Roda, Maria Lioudyno, Evgueni Sevrioukov, Ana María Lacosta, Jesús Canudas, Raquel Sánchez‐Valle, Laura Molina, Iban Aldecoa, Alberto Rabano, Maria Dolores Capilla‐Lopez, Carlos A Saura, Jorge A Busciglio, Juan Fortea, Victor Guallar, Ellen Gelpi, Alberto Lleó

**Affiliations:** ^1^ Sant Pau Memory Unit, Hospital de la Santa Creu i Sant Pau, Institut de Recerca Sant Pau ‐ Universitat Autònoma de Barcelona, Barcelona, Spain; ^2^ Center for Biomedical Investigation Network for Neurodegenerative Diseases (CIBERNED), Madrid, Spain; ^3^ Nostrum Biodiscovery S.L., Protein Engineering Department, Barcelona, Spain; ^4^ University of California Irvine, Institute for Memory Impairments and Neurological Disorders (UCI MIND), Irvine, CA, USA; ^5^ Araclon Biotech‐Grifols, Zaragoza, Spain; ^6^ Alzheimer's disease and other cognitive disorders Unit. Hospital Clínic de Barcelona; FRCB‐IDIBAPS; University of Barcelona, Barcelona, Spain; ^7^ Neurological Tissue Bank of the Biobank ‐ Hospital Clínic ‐ FRCB ‐ IDIBAPS, Barcelona, Spain; ^8^ Pathology Department, Biomedical Diagnostic Center, Hospital Clínic de Barcelona, Barcelona, Spain; ^9^ Reina Sofia Foundation Alzheimer Centre, CIEN Foundation, ISCIII, Madrid, Madrid, Spain; ^10^ Institut de Neurociències, Department de Bioquímica i Biologia Molecular, Facultat de Medicina, Universitat Autònoma de Barcelona, Barcelona, Spain; ^11^ Center for Biomedical Investigation Network for Neurodegenerative Diseases (CIBERNED), Madrid, Madrid, Spain; ^12^ Barcelona Supercomputing Center (BSC), Barcelona, Spain; ^13^ Division of Neuropathology and Neurochemistry, Department of Neurology, Medical University of Vienna, Vienna, Austria; ^14^ CIBERNED, Network Center for Biomedical Research in Neurodegenerative Diseases, National Institute of Health Carlos III, Madrid, Spain

## Abstract

**Background:**

Some studies have described that Amyloid Precursor Protein (APP) C‐terminal fragments (CTFs) accumulate in the brain of patients with Alzheimer's disease (AD). These data rely mostly on biochemical techniques, but the neuroanatomical distribution in human AD brain remains unknown. In this work, we generated a novel APP antibody to investigate the morphological distribution of APP‐CTF accumulation in postmortem human brain.

**Method:**

Cross‐sectional neuropathological study. Formalin‐fixed paraffin‐embedded (FFPE) post‐mortem human brain samples from the hippocampus, frontal and temporal cortex were obtained from the Neurological Tissue Bank (IDIBAPS‐Hospital Clinic Barcelona). The study group consisted of 48 individuals: 10 cases with neuropathological criteria of early‐onset SAD, 12 with ADAD (8 *PSEN1*, 2 *APP* mutation carriers, 2 APP duplications), 5 with DS, 10 with DS‐AD, 7 controls without neurodegenerative pathology and 4 individuals with other neurodegenerative diseases (1 sFTLD‐TDP, 1 *C9orf72*‐FTLD‐TDP, 1 DLB and 1 ALS‐TDP). Antibody generation: APP1B was obtained from 8‐10‐week female BALB/c mice immunized with a C‐terminal APP peptide. Selected hybridomas were cloned and stable clones producing antibodies were expanded. The antibodies were purified using protein G affinity chromatography. Cell culture: Human fetal tissue was obtained to generate primary human cortical culture derived from DS fetuses. A commercially available stable cell line expressing APP‐C99‐GFP was used for immunocytochemistry experiments.

**Result:**

We generated and characterized a novel murine antibody (APP1B) against the C‐terminus of APP. APP1B showed specificity for APP‐CTF in primary human cortical cell culture obtained from DS fetuses and a cell line expressing human APP‐C99. Immunohistochemistry experiments with APP1B antibody in human brain revealed extensive intracellular “tangle‐like” neuronal APP (iAPP) immunoreactivities in AD‐vulnerable areas in sporadic, autosomal dominant AD and in Down syndrome with AD (Figure 1). The immunoreactivity pattern was absent in controls without pathology, DS without AD or other neurodegenerative conditions. iAPP pathology did not colocalize with tau or ubiquitin but followed the neuroanatomical distribution of neuronal degeneration in AD (Figure 2).

**Conclusions:**

We propose iAPP pathology as an unrecognized core neuropathological hallmark of AD. Due to the topographical distribution and selective neuronal involvement, iAPP pathology may represent a link between APP dyshomeostasis and progressive tau aggregation.